# Pressurized Liquid Extraction for the Production of Extracts with Antioxidant Activity from Borututu (*Cochlospermum angolense* Welw.)

**DOI:** 10.3390/foods12061186

**Published:** 2023-03-11

**Authors:** Honória S. Chipaca-Domingos, Federico Ferreres, Tiziana Fornari, Angel Gil-Izquierdo, Benevides C. Pessela, David Villanueva-Bermejo

**Affiliations:** 1 Department of Production and Characterization of Novel Foods, Institute of Food Science Research CIAL (CSIC-UAM), Section of Food Sciences, Faculty of Science, Autonomous University of Madrid (UAM), CEI UAM+CSIC, C/Nicolás Cabrera 9, Campus de Cantoblanco, 28049 Madrid, Spain; 2 National Center for Scientific Research (CNIC/MESCTI), Avenue Ho-Chi-Minh 201, Luanda 34, Angola; 3 Department of Food Technology and Nutrition, Molecular Recognition and Encapsulation (REM) Group, Universidad Católica de Murcia, UCAM, Campus Los Jerónimos, s/n., 30107 Murcia, Spain; 4 Research Group on Quality, Safety and Bioactivity of Plant Foods, Department of Food Science and Technology, CEBAS-CSIC, University Campus of Espinardo—Edif. 25, 30100 Espinardo, Spain; 5 Department of Food Microbiology and Biotechnology, Institute of Food Science Research CIAL (CSIC-UAM), C/Nicolás Cabrera 9, Campus de Cantoblanco, 28049 Madrid, Spain; 6 Higher Polytechnic Institute of Technologies and Sciences, ISPTEC, Av. Luanda Sul, Rua Lateral Vía S10, Talatona, Luanda P.O. Box 1316, Angola

**Keywords:** *Cochlospermum angolense*, borututu roots, pressurized liquid extraction (PLE), antioxidant activity, phenolic compounds

## Abstract

Borututu (*Cochlospermum angolense* Welw.) roots have been described as a rich source of phenolic compounds. Despite the potential of this plant for the production of bioactive extracts, studies reported until now have been scarce, and they have been based on the use of inefficient conventional extraction techniques. In this study, pressurized liquid extraction (PLE) was investigated for the production of borututu root extracts. Different temperatures (50–200 °C) and solvents (water, ethanol, and 50% ethanol:water) were applied. The total phenolic compound (TPC) content, the main phenolic compounds and the in vitro antioxidant activity of the extracts were evaluated. The results were compared with those obtained by conventional decoction with water. The highest concentrations of TPC and antioxidant activity were obtained with 50% ethanol:water, followed by water. The extract obtained with 50% ethanol:water at 150 °C had a TPC concentration of 343.80 mg/g and presented the largest antioxidant activity (1488 and 4979 µmol Trolox/g extract, determined by DDPH and ABTS assay, respectively). These values were considerably higher than those obtained by conventional decoction. Ellagic acid, and ellagic and methyl ellagic acid glycosides were the main phenolic compounds found in the extracts. Therefore, was PLE demonstrated to be a selective and efficient technique to obtain extracts with high concentrations of phenolic compounds and high antioxidant activity form borututu roots.

## 1. Introduction

Plants are a source of natural compounds with bioactive properties, which can be used for the development of products for therapeutic purposes. Africa is one of the continents that is gaining more relevance due to the richness of its flora. Plants of the Cochlospermaceae family are widely distributed in tropical regions [1]. Plants of this genus, such as *Cochlospermum regium*, *Cochlospermum tinctorium*, *Cochlospermum planchonii* and *Cochlospermum angolensis* have been commonly used in traditional medicine [2,3,4].

Borututu (*Cochlospermum angolense* Welw.) is a tree native to Africa. The infusion obtained from borututu roots by decoction with water has been traditionally consumed by many African communities for the treatment of malaria and due to its hepatoprotective properties [5,6]. Borututu has been described as a promising source of bioactive compounds, such as mono- and sesquiterpenes [7], apocarotenoids [8] and phenolic compounds, with the latter group of compounds being related to the majority of bioactive effects of this plant.

The extracts obtained from borututu roots have been reported in the literature due to their potential benefits for health, such as antioxidant, antitumoral [9], antiviral [10] and neuroprotective [11] properties, among others. Costa et al. [12] studied the phenolic compound profile and radical scavenging activity of beverages produced from commercial plants and food products, including borututu, prepared following manufacturer instructions. The concentration of phenolic compounds and the scavenging activity of borututu infusion was low compared to the other products. Nevertheless, these results were attributed to the low amount of plant recommended by the manufacturer to prepare the infusion. Likewise, Barreira et al. [13] investigated the antioxidant effect of beverages prepared from borututu roots and other plants using different commercial formulations and preparation methods. Borututu presented the lowest antioxidant activity, but the results were significantly affected by the preparation method. Ferreres et al. [11] obtained a hydromethanolic and a water extract with a strong antioxidant activity, which was higher than that of ascorbic acid, and a considerable anti-depressant activity. Pereira et al. [9] evaluated the antioxidant and anti-hepatocellular carcinoma activities of commercial syrups from artichoke, milk thistle and borututu roots. Borututu and milk thistle syrups presented the highest antioxidant activity, and the syrups obtained from the extraction of the three combined plants showed hepatoprotective effects. In a further study, Pereira et al. [14] investigated the addition of chestnut honey to artichoke, milk thistle and borututu infusions to potentiate the antioxidant and hepatoprotective properties of these three plants. In general, the addition of honey to the infusions produced a synergistic effect on the antioxidant activity and a lower hepatotoxicity. In another study, Pereira et al. [15] evaluated the phenolic composition and antimicrobial activity of commercial infusions, pills and syrups from borututu. Infusions exerted a good antimicrobial activity against *E. coli*, *E. coli* spectrum extended producer of β-lactamases, *MRSA*, *P. aeruginosa* and methicillin-resistant *S. aureus*, whereas the pills and syrups revealed much less activity.

Despite the potential of borututu roots as a source of antioxidant compounds, the studies reported so far have been mainly based on the use of conventional decoction with water, which is a solvent- and time-consuming extraction technique, whereas the use of cutting-edge and efficient extraction techniques has not been explored yet for the production of extracts from this plant with high antioxidant activity.

Pressurized liquid extraction (PLE) is an efficient extraction technique that has gained much attention for the production of extracts from food and plants [16]. PLE is based on the use of high extraction temperatures, which are above the normal boiling point of the solvents commonly used in extraction processes. For this purpose, pressure is applied to maintain the solvents in a liquid state during the extraction time. The high extraction temperature increases the solubility of compounds and the mass transfer rate and decreases the viscosity and surface tension of the solvents, which lead to fast extraction processes, where high extraction yields can be obtained using small amounts of solvents [17].

PLE has been satisfactorily applied to obtain extracts with high antioxidant activity from plants. For example, Villanueva-Bermejo et al. [18] studied the extraction of caffeine and catechin from green tea using ethyl lactate. The concentration and yield in catechins, which are compounds widely recognized for their antioxidant properties, obtained at 100 and 150 °C were considerably higher than those obtained at lower temperatures. Lopez-Padilla et al. [19] obtained extracts from mortiño using 50% aqueous ethanol at 200 °C with an antioxidant activity 38% higher than that obtained at 50 °C with the same solvent. Sánchez-Martínez et al. [20] studied the extraction of terpenoids from orange juice by-products using PLE with ethyl acetate. The authors obtained an extract at 100 °C with higher antioxidant activity and neuroprotective potential than that obtained at 62.5 °C and ambient temperature. Nieto et al. [21] determined 120 °C to be the optimal temperature using 30% ethanol:water to obtain an extract with high concentration of phenolic compounds and high antioxidant activity from grape stems.

Based on this background, the objective of this study was to investigate the PLE technique for the production of extracts with high antioxidant activity from borututu roots.

## 2. Materials and Methods

### 2.1. Sample Preparation and Chemicals

Absolute ethanol, sodium carbonate and the Folin–Ciocalteu reagent were purchased from Panreac (Barcelona, Spain). Acetonitrile (HPLC grade), formic acid (≥97% purity), ellagic acid (≥95%), gallic acid (≥98%) and protocatechuic acid (97%) standards; 2,2′-Azino-bis(3-ethylbenzothiazoline-6-sulfonic acid (ABTS), 2,2-Diphenyl-1-picrylhydrazyl (DPPH) and Trolox (6-hydroxy-2,5,7,8-tetramethylchroman-2-carboxylic acid) were purchased from Sigma-Aldrich Chemie GmbH (St. Louis, MO, USA).

Borututu roots (*Cochlospermun angolense* welw.) were picked from the northern region of Angola. The roots were sun-dried and ground with a knife mill (Retsch Grindomix GM 200. Retsch GmbH, Haan, Germany). Ground samples were finally sieved (CISA Cedacería Industrial S.L. Barcelona, Spain) prior to extraction to obtain a particle size lower than 250 µm for the samples.

### 2.2. Pressurized Liquid Extraction (PLE)

PLE was carried out using an accelerated solvent extraction unit ASE 350 (Dionex Corporation. Sunnyvale, CA, USA). The extractions were performed at temperatures in the range of 50–200 °C and using water, ethanol and 50:50 ethanol:water (*v/v*) as solvents. The extraction time was 10 min and the pressure applied was 10 MPa.

The extraction cells (10 mL capacity) were loaded with 1 g of sample and mixed with 1 g of sea sand (used as dispersant). The cells were placed in an oven, filled with the solvent and heated up to the desired temperature. Once the temperature was stabilized, the extraction time started (static extraction mode). Then, the cells were washed with fresh solvent, the system was finally purged using nitrogen (90 s) until its complete depressurization, and the extracts were collected in vials.

In addition to PLE, conventional decoction using water was carried out based on the method reported by Pereira et al. [15]. The borututu infusion was prepared by adding 1 g of plant to 200 mL of boiling distilled water. The extract was obtained by filtration after 5 min extraction time.

Water and ethanol were removed from the extracts by freeze-drying and rotary evaporation, respectively. The extracts obtained were stored at −18 °C in darkness until their analysis. All the extractions were carried out in triplicate.

### 2.3. Total Phenolic Compound (TPC) Content

The TPC content was determined in the extracts obtained by PLE and decoction using the Folin–Ciocalteu colorimetric method [22]. Distilled water (600 µL) was mixed with 10 µL of extract or standard solution at a specific concentration and 50 µL of Folin–Ciocalteu reagent. The content was thoroughly mixed and allowed to stand for 1 min. Subsequently, 150 µL of Na_2_CO_3_ (20% *m*/*v*), followed by 190 μL of distilled water, were added, and the mixture was allowed to stand for 2 h at ambient conditions in darkness. The absorbance was measured at 760 nm in a spectrophotometer (UV Vis Genesys™ 10S, Thermo Fisher Scientific, Waltham, MA, USA). Gallic acid was used as a standard, and the results were expressed as GAE (mg of gallic acid/g of extract). The measurements were carried out in triplicate for each extract and gallic acid solution.

### 2.4. Characterization of the Phenolic Profile

Chromatographic analyses were carried out in an HPLC-DAD-ESI(Ion Trap)-MS^n^ unit, as described by Ferreres et al. [23]. The mobile phase consisted of water–formic acid (1%) (A) and acetonitrile (B), starting with 10% B and using a gradient to reach 25% B at 20 min and 65% B at 25 min. The flow rate was 0.8 mL/min, and the injection volume was 20 µL. Chromatograms were recorded at 250, 280 and 340 nm, and the full scan mass covered the range from *m/z* 100 to 1500.

The determination of the exact mass was carried out by UPLC-ESI-QTOF-MS^2^ using an Agilent 1290 Infinity LC system coupled with the 6550 Accurate-Mass QTOF (Agilent Technologies, Waldbronn, Germany) with an electrospray interface (Jet Stream Technology). A Kinetex C18 column (2.1 × 50 mm, 1.7 µm) (Phenomenex. Macclesfield, UK) with a SecurityGuard ULTRA Cartridge of the same material was used, and the analyses were carried out according to the method of Garcia et al. [24]. The composition of the mobile phase was (A) 0.1% (*v/v*) formic acid in water, and (B) 0.1% (*v/v*) formic acid in acetonitrile. The temperature of the column was maintained at 30 °C during the analysis, and compounds were separated using the following mobile phase gradient: initial, 10% B; 12 min, 30% B; 15 min, 50% B. The flow rate applied was 0.5 mL/min. The volume of sample injected was 2 µL. Mass spectra were recorded under the negative ionization mode, and the optimal conditions for the electrospray interface were the following: drying gas temperature, 280 °C; drying gas flow rate, 11 L/min; nebulizer gas pressure, 45 psi; sheath gas temperature, 400 °C; and sheath gas flow rate, 12 L/min. The full scan spectrum was obtained in the range of *m/z* 100–1500. The rest of the conditions were the same as those described by Garcia et al. [24].

### 2.5. Antioxidant Activity

The antioxidant activity of the extracts was determined by the ABTS and DPPH radical scavenging assays. The ABTS^•+^ assay was carried out according to the method reported by Re et al. [25]. The radical cation ABTS^•+^ was generated after reaction of 7 mmol/L ABTS solution and 2.45 mmol/L potassium persulfate and incubation at ambient temperature for 16 h in darkness. The absorbance of ABTS^•+^ radical formed was adjusted to 0.70 ± 0.02 at 734 nm using ethanol (for extracts obtained with ethanol) or PBS (for extracts obtained with water and aqueous ethanol). A volume of extract (10 µL) was incubated with 990 µL of ABTS^•+^ formed as described previously. The reaction took place at ambient temperature for 70 min in darkness, until the absorbance at 734 nm reached a plateau (reaction time experimentally determined).

The DPPH assay was carried out following the method of Brand-Williams et al. [26] with some modifications. First, a stock solution of DPPH radical was prepared by dissolving the radical in methanol and adjusting the solution to 0.7 absorbance. For the assay, 25 µL of extract were mixed with 975 µL of DPPH radical, and the mixture was incubated in darkness at ambient temperature for a specific time (70 min), which was experimentally determined. After that time, the absorbance was determined at 516 nm.

Different concentrations of each extract were assayed, and the EC50 (µg/mL) value was determined. The synthetic radical Trolox was used as a standard, and the results were expressed as TEAC values (mmol Trolox equivalent/g extract). All the experiments were carried out in triplicate.

### 2.6. Statistical Analysis

The results were analyzed statistically by analysis of variance (ANOVA) followed by Tukey´s test (*p* ≤ 0.05,) using the general linear model procedure of the SPSS 26.0 statistical package (SPSS Inc., Chicago, IL, USA).

## 3. Results and Discussion

### 3.1. PLE of Borututu and TPC Content of the Extracts

The PLE of borututu roots was carried out using three different solvents at temperatures from 50 to 200 °C. For comparison purposes, a conventional decoction process was also performed, which is traditionally used by the communities for the consumption of this plant. The total extraction yields obtained are presented in Table 1. As expected, the extraction yields significantly increased with the temperature for the three solvents. At 200 °C, the highest value (57.32%) was obtained with water. The extraction yields also increased with the polarity of the solvent, so the higher the polarity the higher the yield obtained. No significant differences were observed in yields obtained with water and 50% ethanol at temperatures up to 100 °C (18.72–18.99% with aqueous ethanol and 17.94–19.25% with water), but at higher temperatures, the extraction yields achieved with 50% ethanol:water were considerably lower than those obtained with water (26.85–46.29% with 50% ethanol and 43.63–57.32% with water). Therefore, the wider range of polarity of this solvent caused by the addition of ethanol to water did not lead to an increment in the extraction yield compared to the use of neat water. It can be concluded from these results that borututu roots present a high concentration of water-soluble compounds.

The use of PLE allowed obtaining an extraction yield 2.3 times higher than that obtained by conventional decoction (Table 1). This increment in yield was particularly noteworthy when water temperature increased from 100 to 150 °C (yield almost doubled with respect to that obtained by decoction). Therefore, PLE was demonstrated to be an efficient extraction technique for the production of borututu extracts with high extraction yields.

The solvent used, the temperature applied and their interaction significantly affected the concentration of TPC in the extracts (Table 1). The highest values were achieved in the extracts obtained with 50% ethanol:water at all the temperatures explored, followed by those obtained with water. The highest concentration (374.11 mg/g) in the extract obtained with 50% ethanol was achieved at 100 °C. The extracts obtained with water presented intermediate TPC concentrations. On the contrary, the lowest concentrations were obtained with ethanol, especially at 50 °C (72.49 mg/g). The higher TPC concentrations obtained with 50% ethanol may be caused by the larger range of polarity covered by this solvent composition that favors the solubilization of phenolic compounds, compared to that of neat water or ethanol. The high selectivity of hydroalcoholic mixtures for the extraction of phenolic compounds has been thoroughly reported in the literature for other plants and extraction techniques [27,28,29]. In comparison to the extract obtained by decoction with water (175.23 mg/g), the use of PLE and the presence of ethanol in the solvent mixture enabled reaching higher concentrations of TPC in the extracts.

Regarding the effect of temperature, the extraction behavior was different depending on the extraction solvent. In the case of water, no significant differences in the TPC content were observed at 50–100 °C (214 mg/g), and the concentration slightly decreased at 150 and 200 °C (173.02 and 171.65 mg/g, respectively). The concentration of TPC notably increased from 50 °C (263.98 mg/g) to 100 °C (374.11 mg/g) using aqueous ethanol, and it decreased at higher temperatures, especially when the extraction was carried out at 200 °C (208.11 mg/g). In the case of ethanol, the concentration increased with temperature, obtaining the highest value at 200 °C (152.81 mg/g). In extraction processes, it is not only important to obtain an extract concentrated in the compounds of interest, but it is also desirable to obtain high extraction yields of these compounds. The extraction yield of TPC (mg TPC/g feed) increased with the extraction temperature. These high yields were mainly driven by the large total extraction yields obtained at the high temperatures achieved in PLE, rather than by a high concentration of TPC in the extracts. The highest yield of TPC was obtained with water at 200 °C (98.36 mg/g feed). Nevertheless, the yield obtained with 50% ethanol:water at 150 °C was very similar (92.31 mg/g feed), and it should be noted that the TPC concentration with 50% ethanol:water at that extraction temperature was high (343.80 mg/g extract) and close to the largest concentration achieved in this study (374.11 mg/g extract, using 50% ethanol:water at 100 °C). Therefore, 50% ethanol:water is postulated as the best condition among those studied to obtain extracts with a high concentration and extraction yield of TPC.

The concentrations of TPC obtained in the present study using PLE with water and aqueous ethanol were considerably higher than that reported in the literature by conventional extraction with water from borututu (*Cochlospermum angolense*) roots (132.26 mg/g) [6] and similar to that obtained by decoction (175.23 mg/g) in the present study (Table 1). In comparison with other *Cochlospermum* species, borututu has been little-studied. Lamien-Meda et al. [1] carried out an infusion with boiling water for 30 min and ultrasound-assisted extractions at ambient temperature for 1 h using organic solvents (methanol, ethanol, dichloromethane and acetone) from *C. planchonii* and *C. tinctorium* roots. The highest concentration of TPC reported (obtained with methanol) was only 45.6 mg/g. Dall’Acqua et al. [30] obtained extracts from *C. planchonii* with methanol, exploring several extraction techniques (homogenization, ultrasound-assisted extraction, maceration and Soxhlet extraction) and applying different temperatures and extraction times. TPC concentrations ranged from 187.5 mg/g (maceration) to 221.8 mg/g (homogenization), so they were below those obtained in the present study from borututu roots using PLE.

In another study with *C. planchonii* [31], sequential extractions (24 h under stirring at ambient temperature) using solvents with different polarity (dichloromethane, ethyl acetate, ethanol and water) were explored. Ethanol fraction had a TPC concentration of 476 mg/g, which was 27% higher than that obtained in this study using PLE at 100 °C with aqueous ethanol. Nevertheless, unlike the extract obtained by PLE, the process applied by the authors [31] required several extraction stages, long extraction times and high solvent-to-feed ratios (in addition to the use of dichloromethane, which is a toxic and pollutant solvent) resulting in a nonviable process at the industrial scale.

### 3.2. Antioxidant Activity

The antioxidant activity (µmol Trolox/g extract) of the extracts was determined in vitro by the DPPH and ABTS assays (Table 2). With respect to the DPPH method, the values obtained were, in general, similar for the three solvents, although significant differences were found between the solvents and temperatures applied. No significant differences were obtained in the antioxidant activity of the extracts obtained with aqueous ethanol and water, except in those obtained at 50 °C, in which the antioxidant activity obtained with aqueous ethanol was slightly higher. In the case of ethanolic extracts, their antioxidant activity was slightly lower than in those obtained with 50% aqueous ethanol at temperatures from 50 to 150 °C, whereas no significant difference was found in the extracts obtained with both solvents at 200 °C. The extracts with the greatest antioxidant activity were obtained with 50% ethanol and water at 150 °C (1488 and 1413 µmol/g, respectively), and ethanol at 200 °C (1386 µmol/g). The antioxidant activity increased with temperature up to 150 °C for extracts obtained with water and aqueous ethanol and then slightly decreased at 200 °C. In the case of ethanol extracts, the antioxidant activity increased with temperature up to 150 °C, and the activity remained stable at 200 °C. Therefore, the use of temperatures above the boiling point of theses solvents that PLE allows, which cannot be applied in conventional extraction techniques, led to obtaining extracts with the highest antioxidant activity.

Regarding the antioxidant activity determined by the ABTS method (Table 2), the extracts obtained with 50% ethanol showed the highest activity, followed by those of water, whereas the lowest antioxidant activity was obtained in ethanol extracts. As in the DPPH assay, the highest antioxidant activity was obtained with 50% ethanol at 150 °C (4979 µmol/g). This value was 47% and 19% higher than that obtained at 50 °C and 100 °C with the same solvent, respectively; thus, the application of high temperatures favored the production of extracts with greater antioxidant activity. In the case of water extracts, the antioxidant activity of the extract obtained at 150 °C (3645 µmol/g) was 37% lower than that obtained with aqueous ethanol. The ethanol extracts presented the lowest antioxidant activity. Again, the increase in temperature up to 150 °C brought about an increase in the antioxidant activity in extracts obtained with aqueous ethanol and water, followed by a reduction of this capacity at 200 °C. In the case of ethanol, the antioxidant activity increased with temperature, and the highest value with this solvent was obtained at 200 °C (1693 µmol/g).

Based on the results obtained by decoction with water (1179 µmol/g and 1698 µmol/g, determined by the DPPH and ABTS methods, respectively), the antioxidant activity of this extract was 20% (DPPH) and 115% (ABTS) lower than that of the extract obtained by PLE at 150 °C with the same solvent. Compared to the results obtained with PLE using 50% ethanol at 150 °C, the differences with the decoction extract were even higher (26% and 293%, respectively). Therefore, the use of PLE, especially using 50% ethanol:water as solvent, allowed obtaining extracts with an antioxidant activity that could not be achieved with the conventional decoction technique.

The antioxidant activity of borututu and other Cochlospermum species has been mainly attributed to the phenolic compound fraction [3,6]. Pereira et al. [6] reached a TEAC value of 1065 µmol/g (determined by DPPH) in a water extract obtained by infusion from borututu roots. This result was similar to that obtained by decoction in the present study (1179 µmol/g), but 33% and 40% lower than those obtained using PLE at 150 °C with water (1413 µmol/g) and aqueous ethanol (1488 µmol/g), respectively (Table 2). Regarding the use of other *Cochlospermum* species, in the study of Lamien-Meda et al. [1], carried out with *C. planchonii* and *C. tinctorium*, the extract obtained by ultrasound-assisted extraction with methanol had the highest antioxidant activity (498 µmol/g), determined by DPPH. This value was three times lower than the highest obtained in the present study by PLE.

### 3.3. Chemical Analysis of the Extracts

**Figure 1:**
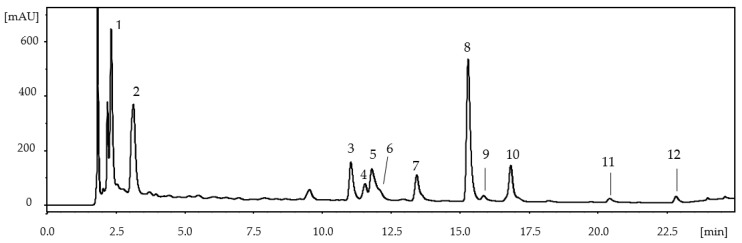
UV-Vis chromatogram (250 nm) of the extract obtained by PLE with water at 100 °C.

The extracts were analyzed by HPLC-DAD/ESI-MS^2^ for their composition in phenolic compounds. The analysis revealed a similar chromatographic profile for all the extracts obtained with the different solvents by decoction and PLE at temperatures between 50 and 150 °C (Figure 1), differing only in the relative abundance of their compounds. A similar behavior was described by Ferreres et al. [11] in extracts obtained by infusion with water and ultrasonic extraction with 1:1 water:methanol, where no differences were observed in the phenolic profile. The extracts obtained at 200 °C presented the same compounds identified at lower temperatures. However, a number of peaks, which were not present in the extracts at 50–150 °C, eluted at 3 min and from 7 to 10 min. These compounds could not be identified, so it was not possible to determine whether they were compounds extracted only at that temperature or degradation products.

With respect to the compounds identified in the extracts, the UV–Vis chromatogram at 250 nm (Figure 1) shows the peaks that, with the exception of **1** and **2**, had characteristic spectra of ellagic acid derivatives (maximum absorbance at ~248–254 nm, shoulders at ~300 and 350 nm and a small maximum at 356–368 nm). This was confirmed using an ellagic acid standard and on the basis of their MS analysis (Table 3), in which a base peak corresponding to the ion of deprotonated ellagic acid (*m/z* 301) or methyl ellagic (*m/z* 315) was observed.

Peak **1** (Table 3) showed an [M-H]- ion at *m/z* 169 and an ion at *m/z* 125 in the MS^2^ spectrum, and Peak **2** presented ions at *m/z* 153 ([M-H]-) and 109 (MS^2^). They were identified as gallic and protocatechuic acid, respectively, by comparison with commercial standards. Peak **5** was found to be ellagic acid ([M-H]- at *m/z* 301, which corresponded with the molecular formula C_14_H_6_O_8_). Peaks **3** and **6** showed deprotonated molecular ions at *m/z* 433 (C_19_H_14_O_12_) and 477 (C_20_H_16_O_12_), respectively, and they were tentatively identified as ellagic acid pentoside (Peak **3**) and ellagic acid rhamnoside (Peak **6**). Both peaks presented in their MS fragmentations a base peak at *m/z* 301, which was attributed to the deprotonated ion of ellagic acid and the loss of a pentose [M-H-132]- and a rhamnose [M-H-146]- moiety. The MS^2^ spectrum of the other compounds (**4** and **7–12**) showed peaks at *m/z* 315, which corresponded to the deprotonated ion of methyl ellagic acid. It was the base peak in compounds **4** and **7–10,** and it was formed from the loss of a hexosyl in Peak **4** [M-H-162]-, a pentosyl in Peaks **7–9** [M-H-132]- and a rhamnosyl radical in Peak **10** [M-H-146]-. Therefore, these compounds were identified as methyl ellagic acid hexoside (**4**), pentoside (**7–9**) and rhamnoside (**10**) isomers. Compounds **11** and **12** presented the same [M-H]- ion at *m/z* 599 (molecular formula C_27_H_20_O_16_). Both compounds showed a MS^2^ ion at *m/z* 447 (C_20_H_15_O_12_) as a result of the loss of a galloyl radical [M-H-152]-, and the deprotonated ion of methyl ellagic acid (*m/z* 315). Based on these results, these compounds were tentatively identified as methyl ellagic acid galloyl pentoside isomers.

The presence of ellagic acid, methyl ellagic acid and their glycosides (mainly hexosides and pentosides) as representative compounds of borututu root extracts has been previously reported [11,15]. Ferreres et al. [11] did not find protocatechuic acid in the extract obtained by infusion with water, as opposed to what has been described in the present and other studies [8,15]. Moreover, the presence of rhamnose and galloyl residues in the ellagic and methyl ellagic acid glycosides has been identified for the first time in this study in borututu extracts. Regarding gallic acid, in addition to the presence of galloyl forms, this compound was also found in its free form in the present study (Table 3), which agrees with the findings of Abourashed and Fu [8], whereas Pereira et al. [15] found this compound attached to the flavanol gallocatechin, thus forming gallocatechin gallate in borututu extracts.

Regarding the possible effect of phenolic compounds on the antioxidant activity, Nergard et al. [3] carried out the fractionation of a *Cochlospermum tinctorium*. The authors obtained a polysaccharide-enriched fraction and observed a reduction in its antioxidant activity, so they indirectly related this bioactivity to the presence of phenolic compounds in the extracts. Ferreres et al. [11] associated the antioxidant activity of a borututu extract with the content of ellagic acid and its derivatives. Abourashed and Fu [8] determined by DPPH assay the antioxidant activity of a methanolic extract from borututu and the fractions obtained by fractionation with different organic solvents. The authors concluded that the highest antioxidant activity observed was produced by gallic and protocatechuic acid, which were isolated with high purity. Their bioactivity was much higher than that obtained in the original methanolic extract and in the gallic and protocatechuic-free fractions obtained. All these organic acids (gallic, protocatechuic and ellagic acid) are present in the extracts obtained in the present study, and this could explain in part the higher TEAC values achieved (Table 2).

## 4. Conclusions

The extracts obtained from borututu (*Cochlospermum angolense* Welw) roots have been associated with different biological activities, such as antioxidant activity, which has been mainly attributed to the phenolic compound fraction and its high content in borututu roots. The studies reported until now have been mainly based on the use of conventional decoction with water and other low-efficiency extraction techniques. In the present study, the PLE of borututu roots has been explored for the production of extracts with high antioxidant activity.

The use of 50% ethanol allowed obtaining extracts with the highest concentration of TPC and antioxidant activity, whereas the lowest values were obtained in ethanol extracts. Regarding the effect of temperature, a high concentration of TPC and the largest antioxidant activity was obtained at 150 °C with 50% ethanol. Even though the highest concentration of TPC was obtained with this solvent at 100 °C (374.11 mg/g), the value achieved at 150 °C (343.80 mg/g) was very similar. Moreover, the antioxidant activity of extracts obtained with 50% ethanol at 150 °C, determined by DPPH and ABTS, was 1488 µmol/g and 4979 µmol/g, respectively. These values were considerably higher than those reported in the literature for extracts from borututu roots and other *Cochlospermum* species obtained by applying conventional extraction techniques.

With respect to the antioxidant activity of the extracts obtained by conventional decoction with water (1179 µmol/g and 1698 µmol/g, determined in the DPPH and ABTS assay, respectively), these values were 20% (DPPH method) and 115% (ABTS method) lower than those obtained with PLE using water at 150 °C. Compared to the extracts obtained with 50% ethanol at 150 °C, the antioxidant activity achieved in the extract produced by conventional decoction was 26% and 293% lower. Therefore, the use of PLE allowed obtaining extracts with high antioxidant activity that could not be achieved with the conventional decoction technique, especially using 50% ethanol as solvent.

The analysis of the extracts by HPLC-DAD/ESI-MS^2^ revealed the presence of gallic acid, protocatechuic acid, ellagic acid, and ellagic and methyl ellagic acid derivatives as the main phenolic compounds identified. Ellagic acid and methyl ellagic acid were mostly in the form of glycosides, especially pentosides, although rhamnose and hexose were also observed in the structures. In addition, methyl ellagic acid galloyl pentosides were also found. All these compounds were present in all the extracts, and, in general, no differences were observed in the phenolic profile at temperatures between 50 and 150 °C for the different solvents used.

## Figures and Tables

**Table 1:** Total extraction yield (g extract/g feed x 100) and TPC (mg/g extract) obtained by PLE and conventional decoction from borututu roots.

Extraction Technique	Solvent	T (°C)	Y (%)	TPC (mg/g)
PLE	Water	50	17.94 ± 2.60 ^d^	213.95 ± 0.87 ^d^
		100	19.25 ± 2.89 ^d^	213.71 ± 0.83 ^d^
		150	43.63 ± 0.10 ^b^	173.02 ± 0.28 ^e^
		200	57.32 ± 0.83 ^a^	171.65 ± 0.59 ^e^
	50% ethanol: water	50	18.99 ± 0.24 ^d^	263.98 ± 0.41 ^c^
		100	18.72 ± 3.29 ^d^	374.11 ± 0.49 ^a^
		150	26.85 ± 0.64 ^c^	343.80 ± 0.49 ^b^
		200	46.29 ± 2.58 ^b^	208.11 ± 0.76 ^d^
	Ethanol	50	4.58 ± 0.27 ^f^	72.49 ± 0.28 ^i^
		100	7.17 ± 0.76 ^e,f^	110.71 ± 0.22 ^h^
		150	10.24 ± 0.54 ^e^	130.97 ± 0.58 ^g^
		200	18.89 ± 1.10 ^d^	152.81 ± 0.10 ^f^
Decoction	Water		24.39 ± 2.1	175.23 ± 0.13

Mean values with different letters in the same column differ significantly (*p* ≤ 0.05).

**Table 2:** Antioxidant activity of the extracts (µmol Trolox/g extract).

Extraction Technique	Solvent	T (°C)	DPPH	ABTS
PLE	Water	50	1208 ± 41 ^d,e,f^	2812 ± 49 ^f^
		100	1286 ± 39 ^c,d^	3475 ± 22 ^d,e^
		150	1413 ± 34 ^a,b^	3645 ± 52 ^c^
		200	1276 ± 39 ^c,d,e^	3590 ± 9 ^c,d^
	50% ethanol: water	50	1358 ± 55 ^b,c^	3387 ± 24 ^e^
		100	1392 ± 14 ^a,b,c^	4174 ± 65 ^b^
		150	1488 ± 27 ^a^	4979 ± 29 ^a^
		200	1284 ± 45 ^c,d^	3599 ± 128 ^c,d^
	Ethanol	50	1108 ± 31 ^f^	639 ± 27 ^j^
		100	1156 ± 35 ^e,f^	1005 ± 25 ^i^
		150	1300 ± 58 ^b,c,d^	1366 ± 42 ^h^
		200	1386 ± 67 ^a,b,c^	1693 ± 59 ^g^
Decoction	Water		1179 ± 2	1698 ± 12

Mean values with different letters in the same column differ significantly (*p* ≤ 0.05).

**Table 3:** Retention time (Rt), molecular formula and main ions generated in the analysis of phenolic compounds from borututu extracts.

Compound	Tentative Identification *	Rt	Molecular Formula	[M-H]^−^ (*m/z*)	MS^2^ (*m/z*)
1	Gallic acid	2.3	C_5_H_5_O_3_	169.0148	125.0242
2	Protocatechuic acid	3.1	C_5_H_5_O_2_	153.0193	109.0293
3	EA pentoside	11.0	C_19_H_14_O_12_	433.0402	300.9966
4	MEA hexoside	11.6	C_21_H_18_O_13_	477.0682	315.0140
5	EA	11.8	C_14_H_6_O_8_	300.9979	
6	EA rhamnoside	12.1	C_20_H_16_O_12_	447.0569	300.9970
7	MEA pentoside	13.4	C_20_H_16_O_12_	447.0570	315.0145
8	MEA pentoside	15.3	C_20_H_16_O_12_	447.0574	315.0142
9	MEA pentoside	16.0	C_20_H_16_O_12_	447.0569	315.0147
10	MEA rhamnoside	16.8	C_21_H_18_O_12_	461.0732	315.0147
11	MEA galloyl pentoside	20.4	C_27_H_20_O_16_	599.0689	447.0567, 315.0146
12	MEA galloyl pentoside	22.8	C_27_H_20_O_16_	599.0691	447.0569, 315.0144

* EA: ellagic acid. MEA: methyl ellagic acid.

## Data Availability

Data are contained within the article.
